# Endothelin-B Receptors and Left Ventricular Dysfunction after Regional versus Global Ischaemia-Reperfusion in Rat Hearts

**DOI:** 10.1155/2012/986813

**Published:** 2012-07-12

**Authors:** Sofia-Iris Bibli, Eleni V. Toli, Agapi D. Vilaeti, Varnavas C. Varnavas, Giannis G. Baltogiannis, Apostolos Papalois, Zenon S. Kyriakides, Theofilos M. Kolettis

**Affiliations:** ^1^Department of Pharmaceutical Chemistry, University of Athens School of Pharmacy, University Campus, Zografou, Athens 15771, Greece; ^2^Cardiovascular Research Institute, Zoodoxos, Ioannina 45500, Greece; ^3^Department of Cardiology, University of Ioannina, 1 Stavrou Niarxou Avenue, Ioannina 45110, Greece; ^4^Department of Cardiology, University Hospital, 55 Hufelandstrasse, Essen 45122, Germany; ^5^Experimental Research Center ELPEN, 95 Marathonos Avanue, Pikermi, Athens 19009, Greece; ^6^Department of Cardiology, Athens Red Cross Hospital, 1 Erythrou Stavrou Street, Athens 11526, Greece

## Abstract

*Background*. Endothelin-1 (ET-1) is implicated in left ventricular dysfunction after ischaemia-reperfusion. ETA and ETB receptors mediate diverse actions, but it is unknown whether these actions depend on ischaemia type and duration. We investigated the role of ETB receptors after four ischaemia-reperfusion protocols in isolated rat hearts. *Methods*. Left ventricular haemodynamic variables were measured in the Langendorff-perfused model after 40- and 20-minute regional or global ischaemia, followed by 30-minute reperfusion. Wild-type (*n* = 39) and ETB-deficient (*n* = 41) rats were compared. Infarct size was measured using fluorescent microspheres after regional ischaemia-reperfusion. *Results*. Left ventricular dysfunction was more prominent in ETB-deficient rats, particularly after regional ischaemia. Infarct size was smaller (*P* = 0.006) in wild-type (31.5 ± 4.4%) than ETB-deficient (45.0 ± 7.3%) rats after 40 minutes of regional ischaemia-reperfusion. Although the recovery of left ventricular function was poorer after 40-minute ischaemia-reperfusion, end-diastolic pressure in ETB-deficient rats was higher after 20 than after 40 minutes of regional ischaemia-reperfusion. *Conclusion*. ETB receptors exert cytoprotective effects in the rat heart, mainly after regional ischaemia-reperfusion. Longer periods of ischaemia suppress the recovery of left ventricular function after reperfusion, but the role of ETB receptors may be more important during the early phases.

## 1. Introduction

Myocardial infarction is the most common clinical manifestation of cardiovascular disease, which remains a leading cause of death worldwide [1]. Prompt restoration of coronary blood flow in the acute phase decreases acute and long-term morbidity and mortality. However, the major limitation of this strategy is reperfusion injury, defined as a second peak of myocardial necrosis, initiated after the onset of reperfusion [2].

Endothelin-1 (ET-1) is a 21-amino-acid peptide, first identified by Yanagisawa and coworkers in 1988 [3]; in addition to its potent vasoconstrictor properties, ET-1 acts also on ventricular myocytes [4]. The effects of ET-1 are mediated via two G-protein-coupled receptors, namely, endothelin-A (ETA) and endothelin-B (ETB), which are abundantly present in the myocardium [5]. ET-1 exerts most of its effects via ETA receptors, whereas the role of ETB receptors in various disease states is incompletely understood [4].

Previous reports have demonstrated elevated circulating ET-1 levels [6–8], as well as increased ET-1 expression in the infarct zone [9], in response to prolonged myocardial ischaemia. ET-1 increases intracellular calcium concentration in ventricular myocytes [10], a well-described factor resulting in cellular necrosis [11]. These effects, along with microvascular obstruction, implicate ET-1 as a mediator of reperfusion injury [12].

Based on the pathophysiologic role of ET-1 during ischaemia-reperfusion, a large number of experimental studies (reviewed in references [12–14]) have investigated the therapeutic potential of ET-receptor blockade in decreasing myocardial necrosis and reperfusion injury and improving left ventricular (LV) dysfunction. However, these studies yielded mixed results, ranging from neutral to remarkably beneficial effects. For example, Dagassan et al. [15] using the dual (ETA and ETB) receptor antagonist bosentan reported no changes in postischaemic LV haemodynamic variables, whereas Lee et al. [16] found a 44% reduction in infarct size after selective ETA receptor blockade. In addition to the variability in ischaemia-reperfusion protocols utilized, the most likely explanation for these discrepant findings is the diversity of agents examined, displaying different degrees of selectivity against ETA and ETB receptors. 

Under normal conditions, ETB receptors mediate the release of vasodilators and contribute to ET-1 clearance from the circulation [17]. However, it remains uncertain whether these beneficial actions are maintained under ischaemic conditions. In 2005, an important study by Yamamoto et al. [18] examined the role of ETB receptors in ischaemia-reperfusion, using the isolated, Langendorff-perfused rat-heart model; they [18] concluded that ETB receptors are beneficial in this setting and ameliorate postischaemic LV dysfunction. However, two methodological issues should be considered in this work. *First*, prolonged ischaemic periods in the Langendorff-perfused rat-heart model result in pronounced and sustained LV dysfunction [19–22], raising some limitations in the study of haemodynamic responses. Moreover, the 40-minute ischaemic period in the Langendorff-perfused model likely corresponds to prolonged ischaemia in the clinical scenario; however, such cases are observed progressively less frequently, given the refinement of emergency medical systems and the widespread use of thrombolysis and/or primary percutaneous coronary interventions [23]. *Second* and foremost, although extensively utilized, the model of zero-flow global myocardial ischaemia presents clear-cut disadvantages with respect to its relevance with acute coronary artery occlusion in patients [20–22].

The purpose of the present study was to further examine the pathophysiologic role of ETB receptors (a) on the infarct size and (b) on LV function after ischaemia-reperfusion. We used the isolated, Langendorff-perfused working heart model, which enables accurate assessment of LV systolic and diastolic function indices. This model is pertinent, as ET-1-release has been demonstrated after ischaemia-reperfusion [24]. As in the study by Yamamoto et al. [18], we used a rat strain, carrying a naturally occurring deletion in the ETB receptor gene that abrogates the expression of functional ETB receptors. 

In the present work, we hypothesized that the effects of ETB receptor activation may vary, depending on the type and duration of ischaemia. For this purpose, we examined LV haemodynamic variables after four ischaemia protocols, namely, after 40- and 20-minute regional or global ischaemia, all followed by 30 minutes of reperfusion. Based on the considerations stated above, the selection of the 40- and 20-minute ischaemia intervals was based on their potential correspondence with delayed or timely (resp.) admission of patients after acute myocardial infarction, whereas the 30-minute-reperfusion interval is widely used in experimental models and enables comprehensive assessment of postischaemic LV dysfunction [20–22]. 

## 2. Materials and Methods

### 2.1. Animal Cohort

The total animal cohort comprised 80 male rats (20–24 weeks of age); previous experience from our laboratory [25–27] indicates that this age range provides favourable experimental conditions, not only in terms of perioperative mortality, but also in terms of heart dimensions. All animals received humane care, according to European legislation (*European Union directive for the protection of animals used for scientific purposes*, 2010/63/EU); they were housed 1-2 per cage, under optimal laboratory conditions (controlled temperature, humidity, and 12 : 12 hour-light : dark cycles), with free access to water and standard rodent chow. 

Of the experimental animal cohort, 39 (20–23 weeks of age, weighing 240 ± 2 g) were wild type and 41 (20–24 weeks of age, weighing 259 ± 3 g) were homozygous ETB deficient. The latter rat model has been characterized previously [28, 29], and a colony is maintained in our animal facilities, kindly provided by Professor M. Yanagisawa (University of Texas Southwestern Medical Center, Dallas, TX, USA). This rat strain carries a 301-bp deletion in ETB receptors, resulting in abnormal mRNA transcript that completely abrogates functional ETB receptor expression [30]. The absence of ETB receptors in this rat strain has been confirmed by polymerase chain reaction, as well as by *in situ* hybridization for ETB mRNA [29]. To prevent premature death of intestinal obstruction in these animals, dopamine *β*-hydroxylase promoter has been used to direct ETB transgene expression and to support normal enteric nervous system development [28]. With this intervention, the animals live into adulthood, but are deficient of ETB receptors in the cardiovascular system, making this rat strain a useful tool in the study of the pathophysiology of ET-1. 

Further characterization of this ETB-deficient rat strain demonstrated markedly increased levels of immunoreactive circulating plasma ET-1 concentrations [29]. Additionally, higher ETA receptor protein expression was shown in homozygous, compared to heterozygous animals [31]; this difference was observed in vascular membrane from small mesenteric arteries, after western blot analysis [31]. Nonetheless, functional uncoupling has been observed between ETA receptor expression and activity [31], which may depend on the vascular bed examined [31, 32]. As noted above, dopamine *β*-hydroxylase promoter control is absent in the cardiovascular system of this rat strain [28, 29]; thus, this “partially rescued” phenotype permits accurate evaluation of ETB receptors in experimental models of myocardial ischaemia. 

### 2.2. Isolated Rat-Heart Preparation

The animals were anaesthetized with an intramuscular injection of ketamine (50 mg/kg) and xylazine (5 mg/kg) and were euthanized by cervical dislocation. The low dosage of this anaesthetic protocol, coupled with its brief duration associated with the *ex vivo *experimental conditions, precludes significant effects on cardiac function. After a left lateral thoracotomy, the hearts were rapidly excised and were mounted on a Langendorff apparatus (ML870B2 system, ADInstruments, Oxfordshire, UK), as previously described [27, 33]. The preparations were perfused at a constant flow of 12.5 ml/min with Krebs-Henseleit solution at the following composition (in mmol/L): NaCl: 118.1, KCl: 4.6, CaCl_2_: 2.5, MgSO_4_: 1.2, KH_2_PO_4_: 1.2, NaHCO_3_: 24.8, and glucose: 10.33. The perfusate was bubbled continuously with a gas mixture of 95% O_2_ and 5% CO_2_. The pH and temperature were kept stable at 7.4 and 37°C, respectively. To ensure comparable measurements, atrial pacing was performed at a rate of 300 beats per minute during the stabilization period and during reperfusion; as in previous reports, pacing was continued during regional [34], but not during global [27, 33] ischaemia. 

### 2.3. Haemodynamic Measurements

A water-filled balloon, connected to a pressure transducer, was placed into the LV through a left atrial incision. The balloon volume was adjusted to achieve a stable LV end-diastolic pressure (EDP) of 10 mmHg. LV pressure signal was continuously recorded and analyzed online by a dedicated software program (Chart 5, version 5.4.2., ADInstruments, Oxfordshire, UK). After a stabilization period of 20 minutes, LV developed pressure (DP), defined as LV systolic minus diastolic pressure, EDP, and maximum positive (maximum +*dp/dt*) and negative (maximum −*dp/dt*) values of the first derivative of pressure were recorded for 30 minutes at 10-minute intervals, as previously published [18]. 

### 2.4. Experimental Protocol

Two series of experiments were performed, namely, global and regional ischaemia. In the first series, both wild-type and ETB-deficient rats underwent either 40 or 20 minutes of global ischaemia, followed by 30 minutes of reperfusion. Zero-flow global ischaemia was attained with turning off the aortic cannula and reperfusion with the restoration of flow. In the second series, both rat strains underwent either 40 or 20 minutes of regional ischaemia, followed by 30-minutes of reperfusion. Regional ischaemia was induced by left coronary artery ligation, as described, [35, 36]. Briefly, the left coronary artery was encircled with a 4–0 suture near its origin, extending from the pulmonary cone to the myocardium under the left atrial appendage; following these anatomical landmarks ensures comparable myocardial ischaemic area in all experiments. The suture was passed through a snare, the tightening and release of which induced ischaemia and reperfusion, respectively. 

### 2.5. Infarct Size

Infarct size was measured in a separate series of experiments after 40 and 20 minutes of regional ischaemia, followed by 30 minutes of reperfusion, in wild-type (*n* = 10) and in ETB-deficient rats (*n* = 11). Infarct size was expressed as a percentage of ischaemic myocardial area at risk, as previously published from our laboratory [33, 37]. The heart was perfused via the aorta with normal saline for 2 minutes, until all residual Krebs-Henseleit solution had been removed from the coronary arteries; subsequently, the coronary ligature was retightened at the same site, and 5ml green-fluorescent microspheres, 2–9 *μ*m in diameter (Duke Scientific Corp., Palo Alto, CA, USA), were infused over 5-minutes. The right ventricle was removed, and the hearts were frozen at −20°C for 24 hours and were sliced into 2 mm-sections from apex to base. Following incubation in 1% triphenyltetrazolium chloride in isotonic phosphate-buffer solution (at 37°C, pH 7.4) for 20 minutes, the slices were immersed in 10% formaldehyde solution for 24 hours. After placement between glass plates, the risk zone, the infarcted area, and the normal myocardium were identified under ultraviolet light (*λ* = 366 nm) and traced on an acetate sheet. These areas were measured with the use of Image Tool (The University of Texas Health Science Center, San Antonio, TX USA); the volumes of infarct area and area at risk were expressed in cm^3^, and the percent ratio of infarct area/area at risk was calculated.

### 2.6. Statistical Analysis

All values are reported as mean ± standard deviation. The infarct size values displayed a normal distribution (as per Kolmogorov-Smirnov test for normality) and were compared with the use of Student's *t*-test. Nonparametric analyses were performed to evaluate differences in haemodynamic variables, since normal distribution was not consistently present. Specifically, changes of haemodynamic variables over time were assessed with Friedman analysis of variance, whereas differences between groups in haemodynamic variables during reperfusion (expressed as percent changes compared to baseline) were examined with the use of Mann-Whitney *U* test. Statistical trend was defined as 0.05 < *P* < 0.1 and statistical significance as *P* < 0.05. 

## 3. Results

**Figure 1:**
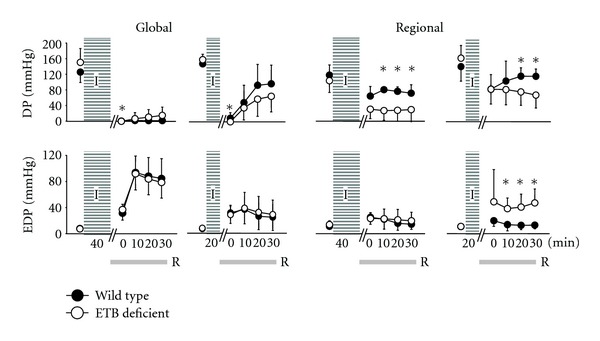
Left-ventricular-developed pressure (LVDP, upper) and end-diastolic pressure (LVEDP, lower) after ischaemia (I)/reperfusion (R) in wild-type (dark dots) and ETB-deficient rats (white dots). Asterisk denotes significant differences between the two groups.

**Figure 2:**
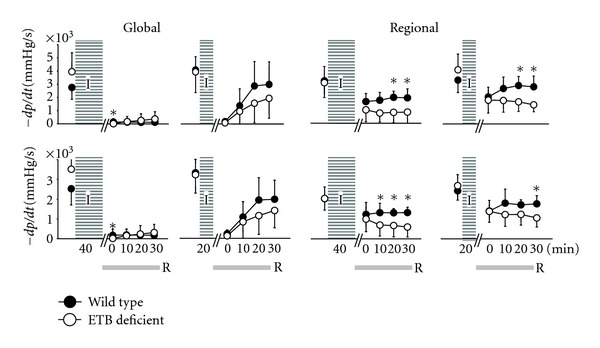
Maximum values (maximum +*dp/dt*, upper) and minimum values (maximum −*dp/dt*, lower) of the first derivative of left ventricular pressure after ischaemia (I)/reperfusion (R) in wild-type (dark dots) and ETB-deficient rats (white dots). Asterisk denotes significant differences between the two groups.

Changes in DP and EDP are graphically depicted in Figure 1 and in the first derivative of LV pressure (maximum +*dp/dt* and maximum −*dp/dt*) in Figure 2.

### 3.1. 40-Minute Global Ischaemia

DP at the onset of reperfusion was lower (*P* = 0.035) in ETB-deficient rats, but subsequent values were comparable between the two groups. No significant differences were found in EDP between groups. Maximum +*dp/dt* and maximum −*dp/dt* were lower at the onset of reperfusion in ETB-deficient rats, but subsequent values were comparable between groups. 

### 3.2. 20-Minute Global Ischaemia

DP at the onset of reperfusion was lower (*P* = 0.046) in ETB-deficient rats, but subsequent values were comparable in the two groups, as were all remaining haemodynamic variables. 

### 3.3. 40-Minute Regional Ischaemia

There was a trend (*P* = 0.086) towards lower DP values in ETB-deficient rats at the onset of reperfusion and this difference became significant (all *P* < 0.03) at 10, 20, and 30 minutes after the onset of reperfusion. EDP did not differ between groups. Maximum +*dp/dt* was lower (both *P* < 0.01) in ETB-deficient rats at 20, and 30 minutes of reperfusion. A similar pattern was seen in maximum −*dp/dt*, with differences (all *P* < 0.03) seen at 10, 20, and 30 minutes of reperfusion. 

### 3.4. 20-Minute Regional Ischaemia

DP was lower (both *P* < 0.03) in ETB-deficient rats at 20 and 30 minutes of reperfusion. EDP was higher (all *P* < 0.02) in ETB-deficient rats at 10, 20, and 30 minutes of reperfusion. Maximum +*dp/dt* was marginally (*P* = 0.054) lower in ETB-deficient rats at 10 minutes and significantly (both *P* < 0.02) lower at 20 and 30 minutes. Maximum −*dp/dt* was significantly (*P* = 0.0373) lower in ETB-deficient rats at the 30th minute of reperfusion.

### 3.5. Infarct Size after Regional Ischaemia-Reperfusion

**Figure 3:**
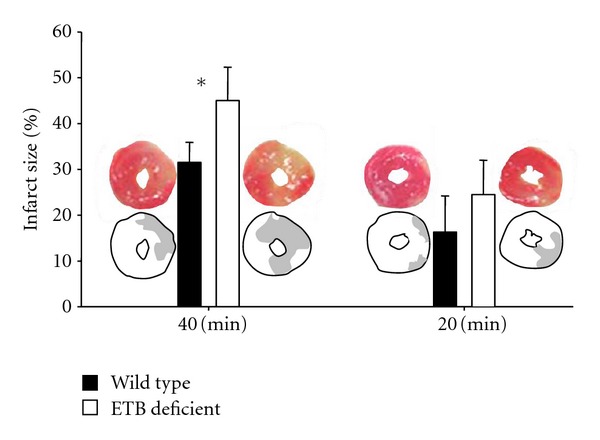
Infarct size (expressed as percent ratio of necrotic area/area at risk) in wild-type (dark bars) and ETB-deficient rats (white bars) after 40- and 20-minute regional ischaemia. Asterisk denotes significant differences between the two groups. Representative examples of infarct size measurements (histological samples and schematic illustrations) are shown adjacent to the bars.

As shown in Figure 3, 40 minutes of regional ischaemia, followed by 30 minutes of reperfusion, resulted in larger (*P* = 0.00609) infarct size in ETB-deficient (45.0 ± 7.3%) than in wild-type (31.5 ± 4.4%) rats. A similar trend (*P* = 0.097) was seen after 20 minutes of regional ischaemia, followed by 30 minutes of reperfusion, with values at 24.5 ± 7.5% in ETB-deficient and 16.3 ± 7.9 in wild-type animals.

### 3.6. Comparison between Regional and Global Ischaemia in Wild-Type Rats

Compared to the 40-minute regional ischaemia-reperfusion protocol, global ischaemia-reperfusion of equal duration in wild-type rats resulted in significantly (all *P* < 0.01) lower DP, maximum +*dp/dt*, maximum −*dp/dt*, and higher EDP values. Similarly, 20 minutes of global ischaemia in wild-type rats resulted in significantly (all *P* < 0.01) higher EDP values, compared to regional ischaemia of equal duration. In contrast, DP was lower (*P* = 0.0019) only at the onset of reperfusion after 20-minute global, compared to regional ischaemia. Maximum +*dp/dt* and maximum −*dp/dt* were lower only at the onset and at the 10th minute of reperfusion. 

### 3.7. Comparison between Regional and Global Ischaemia in ETB-Deficient Rats

Compared to 40 minutes of regional ischaemia, global ischaemia of equal duration in ETB-deficient rats resulted in significantly (all *P* < 0.05) lower maximum +*dp/dt* (with the exception of values observed at the 30th minute), maximum −*dp/dt*, and higher EDP values. In contrast, recovery of DP was equally poor after either 40-minute global or regional ischaemia, with the exception of lower (*P* = 0.0011) values observed at the onset of reperfusion following global ischaemia. The recovery of all haemodynamic variables was comparable after 20 minutes of either global or regional ischaemia, except from values at the onset of reperfusion, when DP, maximum +*dp/dt*, and maximum −*dp/dt* were lower (all *P* < 0.01) after global ischaemia.

### 3.8. Comparison between 40- and 20-Minute Ischaemia in Wild-Type Rats

Compared to the 40-minute protocol, 20 minute global ischaemia-reperfusion was associated with improved recovery of LV function. This difference (both *P* < 0.01) was seen in DP at the 20th, and 30th minutes of reperfusion, in maximum +*dp/dt* (all *P* < 0.05) at the 10th, 20th, and 30th minutes of reperfusion and in maximum −*dp/dt* (both *P* < 0.02) at the 20th and 30th minutes of reperfusion. 

The same pattern of recovery of LV function was observed after 40- and 20-minute regional ischaemia protocols in DP (both *P* < 0.01) at the 20th and 30th minute of reperfusion, in maximum +*dp/dt* (all *P* < 0.05) at the 10th, 20th, and 30th minute of reperfusion and in maximum −*dp/dt* (both *P* < 0.02) at the 20th and 30th minute of reperfusion. 

### 3.9. Comparison between 40- and 20-Minute Ischaemia in ETB-Deficient Rats

Compared to the 40-minute protocol, 20-minute global ischaemia-reperfusion was associated with improved recovery of LV function. This difference was seen in DP, maximum +*dp/dt*, and maximum −*dp/dt* during the entire reperfusion period (all *P* < 0.01) and in EDP at the 10th, 20th, and 30th minutes of reperfusion (all *P* < 0.05). 

These differences were less pronounced in regional ischaemia protocols and were observed in DP [at the 10th minute (*P* = 0.032125), with a trend (*P* = 0.08647) present at the 20th minute of reperfusion]. No significant differences were observed in maximum +*dp/dt* and in maximum–*dp/dt*. Surprisingly, EDP was higher after 20 than after 40 minutes of regional ischaemia; specifically, there was a trend (*P* = 0.06329) towards higher values at the 10th minute, but significant differences were present at the 20th (*P* = 0.02227) and 30th (*P* = 0.00427) minutes of reperfusion.

## 4. Discussion

### 4.1. Main Findings

Despite ample research efforts, the pathophysiology of reperfusion injury after prolonged myocardial ischaemia remains incompletely understood. A substantial body of experimental data [13, 14] has implicated ET-1 as an important mediator in this process; ET-1 acts via ETA receptors, whereas the role of ETB receptors is unclear. Here, we report cytoprotective effects of ETB receptors on the ventricular myocardium after regional ischaemia-reperfusion, evidenced by less myocardial necrosis and improved LV haemodynamic indices in wild-type, compared to ETB-deficient rats. 

### 4.2. Comparison with Previous Studies

Previous studies investigating the role of ETA and ETB receptors after ischaemia-reperfusion produced diverse results, but these should be viewed with caution, given the limitations associated with pharmacologic blockade of ET receptors [12–14]. In contrast, the use of the ETB-deficient rat model (developed and validated at Professor Yanagisawa's laboratory [28, 29]) in our experiments permits more accurate assessment of the pathophysiologic role of ET receptors. Our findings confirm the conclusions on the role of ETA and ETB receptors derived by Yamamoto et al. [18], who used the same ETB-deficient rat strain. However, the main addition of the present work is the evaluation of two important parameters, namely, the type (regional versus global) and duration (40 versus 20 minutes) of ischaemia (all followed by a 30-minute reperfusion period).

### 4.3. Regional versus Global Ischaemia

Zero-flow global ischaemia protocols have been widely used in the *ex vivo* Langendorff-perfused rat-heart model. The main advantage is the comparable and easily reproducible extent of myocardial ischaemia, but the distant relevance with acute coronary artery occlusion in patients constitutes the main disadvantage of this model [20–22].

In the present study, we included protocols of ischaemia, induced by *ex vivo* left anterior descending coronary artery ligation. This technique, originally described in 1975 [38], combines the closer resemblance with myocardial ischaemia/infarction in man, with the accuracy of measurements offered by the *ex vivo* heart model; based on these advantages, this method is considered of value in the investigation of myocardial ischaemia/infarction [20, 21]. Our findings of more physiological values after regional ischaemia, as opposed to the marked postischaemic LV dysfunction, observed mainly after 40-minute-global ischaemia, reinforce these considerations [20, 21]. Moreover, the less prominent LV dysfunction, produced by regional ischaemia, permitted the more accurate evaluation of the effects of ETB receptors on the ventricular myocardium.

In our experiments, postischaemic LV dysfunction after 40 minutes of global ischaemia was more pronounced (in wild-type and in ETB-deficient animals) compared to that reported by Yamamoto et al. [18]. Since heart rate correlates with cardiac work [19], lower heart rate may be cardioprotective [19, 34]; therefore, the difference in postischaemic LV dysfunction between our experiments and those by Yamamoto et al. [18] could be attributed to the effects of higher heart rate in our experiments, produced by atrial pacing at a rate of 300 beats per minute. Indeed, ventricular pacing was shown to enhance postischaemic ventricular dysfunction in this setting [19], although ventricular asynchrony induced by such pacing constitutes a well-described detrimental factor [39].

### 4.4. 40- versus 20-Minute Ischaemia

We evaluated the duration of the ischaemic period, by comparing two commonly used protocols [20–22]. In agreement with previous studies [19], we report exaggerated postischaemic LV dysfunction with 40- more than with 20-minute ischaemia, this difference being more prominent after global than after regional ischaemia. 

The correlation between ischaemia duration (in the ischaemia-reperfusion isolated rat-heart model) and the time to treatment onset (thrombolysis or primary percutaneous coronary intervention) in patients with myocardial infarction is unknown. A recent meta-analysis [40] indicated time intervals from symptom onset to intervention at the range of 90–140 minutes, and these figures appear further improved in more recent programmes [41, 42]. Based on this information, we propose that the 20-minute regional ischaemia protocol applies most to clinical practice, whereas longer ischaemic periods are still relevant but correspond to progressively smaller proportion of patients.

### 4.5. Mechanisms of ETB Receptor-Induced Cytoprotection

The mechanisms of ventricular cytoprotective effects of ETB receptors after ischaemia-reperfusion remain under investigation. ETB receptors may be involved in the local sequestration and clearance of ET-1, not only under normal, but also during ischaemic conditions [43]. Increased ET-1 locally in the myocardium in the chronic absence of ETB receptors may exert vasoconstrictive actions via ETA receptors, thereby increasing coronary artery resistance [43]. Thus, in our experiments, ETA receptor-mediated responses may have been exaggerated in ETB-deficient animals. These notions are reinforced by the experiments of Yamamoto et al. [18], who found exaggerated coronary-flow reduction in ETB-deficient rats, which was restored after selective ETA receptor blockade. In fact, our experiments lend further support to this view, as the differences between wild-type and ETB-deficient rats were relatively minor under conditions of constant coronary flow, in the setting of 40-minute global ischaemia. However, our results point out towards additional underlying mechanisms, based (a) on the different haemodynamic responses still being present after global or (mainly) regional ischaemia (followed by reperfusion) and (b) on the decreased infarct size in wild-type rats after regional ischaemia (followed by reperfusion).

Yamamoto et al. [18] reported enhanced norepinephrine overflow in ETB-deficient rats, suggesting an essential role of ETB receptors in the regulation of local myocardial sympathetic response under ischaemic conditions. This finding was confirmed in a previous study by our group [44], using the *in vivo *model of coronary artery ligation in the same ETB-deficient rat strain; in this study, we found markedly higher sympathetic activation in ETB-deficient rats, assessed by fast Fourier analysis of heart rate variability, as well as by measurement of serum epinephrine and norepinephrine levels [44]. The well-described [45] mechanisms of norepinephrine-induced acceleration of cell death in the ischaemic ventricular myocardium include augmented myocardial oxygen demand, mediated by increased heart rate and contractility, as well as decreased oxygen supply secondary to coronary artery vasoconstriction.

### 4.6. ETB Receptor-Induced Cytoprotection and Ischaemia-Reperfusion Protocols

In our experiments, the differences in haemodynamic variables between wild-type and ETB-deficient rats were less evident after global ischaemia, providing another potential explanation for the discrepant conclusions of previous studies [15, 18] reported in this setting. In contrast, the recovery of left ventricular function was poorer in ETB-deficient rats after regional ischaemia, indicating a pathophysiologic role of ETB receptors in more clinically applicable experimental conditions. 

A paradoxical finding in our experiments was the higher EDP observed in ETB- deficient rats after 20 than after 40 minutes of regional ischaemia. Although difficult to explain, this is in line with our previous observations [44], indicating a prominent role of ETB receptors in sympathetic activation only during the early phase of infarction, with strikingly diminished effects during subsequent stages. 

The time course of norepinephrine release from the sympathetic nerve terminals in the ventricular myocardium in response to prolonged ischaemia has been subject of intense research [46]. During the early phase, this process occurs initially by norepinephrine exocytosis but is subsequently greatly enhanced by reverse-mode function of the norepinephrine transporter, thereby releasing the accumulated free axoplasmic norepinephrine. ET-1 has been shown to promote nonexocytotic norepinephrine release early after prolonged ischaemia [47]; this action occurs via ETA receptor stimulation, resulting in axoplasmic sodium accumulation and reversal of norepinephrine transporter [18, 47]. ETB receptors appear to counteract this effect, evidenced by marked elevation of norepinephrine release after selective ETB receptor blockade in isolated ischaemic guinea pig hearts [48]. However, during subsequent stages, this protective effect of ETB receptors diminishes, possibly due to axoplasmic norepinephrine depletion [44]. 

### 4.7. Strengths and Limitations of the Study

We examined the role of ETB receptors using four ischaemia-reperfusion protocols in the isolated Langendorff-perfused rat heart. The evaluation of ischaemia duration and the induction of clinically relevant ischaemia represent major strengths of our work. However, three limitations should be acknowledged. *First*, coronary flow was held constant throughout the experiments, enabling the assessment of postischaemic LV dysfunction without the confounding factor of coronary autoregulation; nonetheless, the addition of protocols using constant perfusion pressure would have permitted the assessment of the effects of ETB receptors on coronary vascular resistance. *Second*, our study did not address the issue of the pathophysiologic mediators of ETB receptor activation. More specifically, we did not include measurements of norepinephrine levels in the perfusate, that could have expanded previous findings [18, 44]. *Third*, our protocol did not include the calculation of infarct size after global ischaemia-reperfusion; although measurement of diffuse necrosis is more difficult, its addition in the protocol could have increased the value of our results.

## 5. Conclusions

ETB receptors exert cytoprotective effects on the ventricular myocardium after ischaemia-reperfusion in the isolated, Langendorff-perfused working rat heart. These effects are more prominent after regional than after global ischaemia (followed by reperfusion). Longer periods of ischaemia suppress the recovery of LV function after reperfusion, but the role of ETB receptors may be more important during the early phases.
